# Expanding the genotype-phenotype spectrum in SCN8A-related disorders

**DOI:** 10.21203/rs.3.rs-3221902/v1

**Published:** 2023-08-08

**Authors:** Malavika Hebbar, Nawaf Al-Taweel, Inderpal Gill, Cyrus Boelman, Richard A Dean, Samuel J Goodchild, Janette Mezeyova, Noah Gregory Shuart, J. P. Johnson, James Lee, Aspasia Michoulas, Linda L Huh, Linlea Armstrong, Mary B Connolly, Michelle K. Demos

**Affiliations:** Division of Neurology, Department of Pediatrics, BC Children’s Hospital, Faculty of Medicine, University of British Columbia, Vancouver BC; Division of Neurology, Department of Pediatrics, BC Children’s Hospital, Faculty of Medicine, University of British Columbia, Vancouver BC; Division of Neurology, Department of Pediatrics, BC Children’s Hospital, Faculty of Medicine, University of British Columbia, Vancouver BC; Division of Neurology, Department of Pediatrics, BC Children’s Hospital, Faculty of Medicine, University of British Columbia, Vancouver BC; Xenon Pharmaceuticals, 200-3650 Gilmore Way, Burnaby, BC V5G 4W8; Xenon Pharmaceuticals, 200-3650 Gilmore Way, Burnaby, BC V5G 4W8; Xenon Pharmaceuticals, 200-3650 Gilmore Way, Burnaby, BC V5G 4W8; Xenon Pharmaceuticals, 200-3650 Gilmore Way, Burnaby, BC V5G 4W8; Xenon Pharmaceuticals, 200-3650 Gilmore Way, Burnaby, BC V5G 4W8; Division of Neurology, Department of Pediatrics, BC Children’s Hospital, Faculty of Medicine, University of British Columbia, Vancouver BC; Division of Neurology, Department of Pediatrics, BC Children’s Hospital, Faculty of Medicine, University of British Columbia, Vancouver BC; Division of Neurology, Department of Pediatrics, BC Children’s Hospital, Faculty of Medicine, University of British Columbia, Vancouver BC; Department of Medical Genetics, BC Children’s Hospital, Faculty of Medicine, University of British Columbia, Vancouver BC; Division of Neurology, Department of Pediatrics, BC Children’s Hospital, Faculty of Medicine, University of British Columbia, Vancouver BC; Division of Neurology, Department of Pediatrics, BC Children’s Hospital, Faculty of Medicine, University of British Columbia, Vancouver BC

**Keywords:** SCN8A, Developmental and Epileptic Encephalopathy, Epilepsy, Seizure, Electrophysiological study, Variant of Uncertain Significance, Exome sequencing, Loss-of-function, Gain-of-function

## Abstract

**Background:**

*SCN8A*-related disorders are a group of variable conditions caused by pathogenic variations in *SCN8A*. Online Mendelian Inheritance in Man (OMIM) terms them as developmental and epileptic encephalopathy 13, benign familial infantile seizures 5 or cognitive impairment with or without cerebellar ataxia.

**Methods:**

In this study, we describe clinical and genetic results on eight individuals from six families with *SCN8A* pathogenic variants identified via exome sequencing.

**Results:**

Clinical findings ranged from normal development with well-controlled epilepsy to significant developmental delay with treatment-resistant epilepsy. Three novel and three reported variants were observed in *SCN8A*. Electrophysiological analysis in transfected cells revealed a loss-of-function variant in Patient 4.

**Conclusions:**

This work expands the clinical and genotypic spectrum of *SCN8A*-related disorders and provides electrophysiological results on a novel loss-of-function *SCN8A* variant.

## Background

Pathogenic genomic variations in *SCN8A* can cause a spectrum of neurological phenotype characterized by developmental delay, early onset multivariate seizure types, intractable epilepsy, movement disorders and other neurological manifestations.(1–3) Psychomotor development varies from normal to abnormal since birth. Normal development may precede subsequent delay or regression following seizure onset. Variable degrees of intellectual disability is seen with ~ 50% having a severe form. Behavioral abnormalities are also seen in some individuals.

The expression of voltage-gated sodium channels (NaVs) is key for initiation and conduction of action potentials in excitable cells such as skeletal muscle and neurons.(4) Neurons typically express multiple NaV isoforms. Loss-of-function (LoF) and gain-of-function (GoF) of voltage-gated sodium channels can lead to a wide spectrum of phenotypes. *SCN8A* (NaV1.6; OMIM 600702) is one of nine human genes encoding voltage-gated sodium channel α-subunits more recently implicated in epilepsy.(5) *SCN8A* variants in patients with epilepsy primarily result in GoF in Nav1.6 and hyperexcitability of neurons in the central nervous system.(6) Evaluation of the phenotype and genotype spectrum in *SCN8A*-related disorders suggests that GoF mutations are associated with severe epileptic encephalopathy, while LoF mutations cause intellectual disability with or without seizures. Sodium channel-blocking agents are effective on different levels in the treatment of seizures in GoF mutations. Anti-sense oligonucleotide therapy is in clinical trials for GoF variants and several treatment modalities are being explored in research including transfected cell lines and mouse models.(7) Targeted and genome-wide next-generation sequencing (NGS) has significantly increased the number of families identified with *SCN8A*-related disorders, allowing scientists to prioritize functional studies and develop a better understanding of the phenotypic spectrum.(3)

In this case series, we would like to add to the growing clinical and genetic data of over 500 individuals with *SCN8A*-related disorders (8, 9) by reporting 8 affected individuals with variable phenotypes including one family with a previously published variant associated with treatable epilepsy, as well as, novel variants in *SCN8A* identified by exome sequencing. We establish functional evidence for a LoF *SCN8A* variant by using electrophysiological analyses in a patient with intellectual disability, autism spectrum disorder, and abnormal EEG. The patient also presented a co-occurring variant of unknown significance in *KCNQ3*.

## Methods

Six families seen at neurology clinic, British Columbia Children’s Hospital were enrolled in the study. Exome sequencing was performed on the probands. Informed consent was obtained for the use of clinical and research findings for publication. The study has the approval from Institutional Ethics Committee (protocol number H14-01531). Clinical and molecular details of patients are summarized in Table 1. Detailed case description can be found in the Additional file 1.

### Exome sequencing

Exome sequencing was performed in all the families. Detailed methodology and steps followed for exome sequencing wet lab and data analysis has been previously described.(10) Sanger sequencing to validate the variants and to determine the segregation in the families was performed.(11)

#### Functional validation of SCN8A

The functional consequence of the *SCN8A*, c.971G > A (p.Cys324Tyr) variant was examined *in vitro* by heterologous protein expression in Human Embryonic Kidney cells (HEK-293). The electrophysiological properties of the HEK-293 cells expressing the p.Cys324Tyr protein were compared to control cells expressing either the wild-type protein or empty expression vector. Functional studies were not performed for the *KCNQ3* variant in Patient 4.

## Results

**Figure 1:**
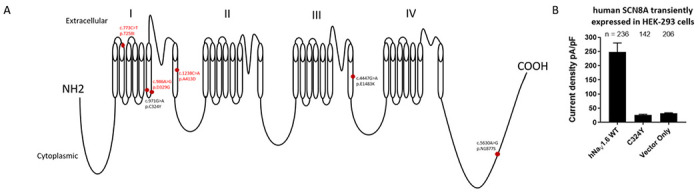
A. Simplified diagram of NaV1.6 channel showing the locations of the variants identified in our cohort (novel mutations are in red font). B. HEK-293 cells were transiently transfected with hNaV1.6 WT, hNaV1.6 C324Y, plasmid vector with no channel construct to look for functional effects of C324Y variant. C324Y current levels were significantly different from WT but not from Vector control.

We studied eight patients from six families (males = 3, females = 5) with *SCN8A* heterozygous mutations. The phenotype ranged from DEE (n = 2), treatment responsive (n = 5) and an unclassified phenotype with possible clinical seizures in Patient 4. The age of seizure onset ranged from 3 months to 10 years. Individuals with DEE and unknown phenotypes presented with profound to severe intellectual disability and severe global developmental delay. Individuals with treatment responsive phenotype were intellectually and developmentally within normal limits. Patient 4 had GDD and autism as a primary clinical phenotype with a characteristic EEG abnormality, with possible clinical seizures, treated with valproic acid which had improved EEG characteristics in the past. Four of them are seizure-free on monotherapy of carbamazepine and one with topiramate and clobazam. Exome sequencing identified three known and three novel heterozygous missense variations in *SCN8A*. Patient 4 also had a heterozygous, *de novo*, missense VUS in *KCNQ3*. Functionally, we observed a LoF, two GoF and three unclassified *SCN8A* variants. Electrophysiological analyses of the *SCN8A* variant in transfected cells revealed a LoF effect in Patient 4 (Fig. 1.).

## Discussion

*SCN8A* variants typically result in a moderate-severe epileptic encephalopathy, and account for 1% of the childhood epileptic encephalopathies.(1) The median age of seizures onset is typically 5 months (range: postnatal day 1 to 18 months of age) with multiple seizure types. The majority of affected patients have mild to severe global developmental delay, abnormal tone, and abnormal movements may be present.(12) In our cohort of eight individuals from six families with *SCN8A*-related disorders, we observed an age of onset ranging from 3 months to 10 years with severe to no clinical seizures. Developmental outcomes varied from profound developmental delay with intellectual disability and behavioural abnormalities to normal development. Developmental delay and age of onset of seizures did not seem to have a correlation in our cohort.(13) The seizure semiology in *SCN8A*-related disorders is variable, including focal seizures, tonic-clonic seizures, epileptic spasms, clonic seizures, absence, and myoclonic seizures.(12, 14) Patients with *SCN8A* mutations also have a high incidence of Sudden Unexpected Death in Epilepsy (SUDEP).(15, 16) We noted a seizure course ranging from self-resolving focal seizures to Lennox-Gastaut syndrome (LGS) manifesting impaired awareness seizures, atypical absence seizures, generalized tonic-clonic seizures, epileptic spasms, and non-convulsive status epilepticus. The most common seizure type has been focal seizures as observed in the earlier reported patients.(17)

The three novel mutations are missense substitutions located on highly conserved transmembrane domains 1 and 2 of NaV1.6 (Fig. 1.). *SCN8A* gene variants causing substitution of amino acid residues in the highly conserved regions are often deleterious.(1) Three mutations (those of Patient 2(18, 19), Patient 3(20), and Patient 4(21)) were described previously. The clinical features of patient 2, and 3 were quite similar as the characterization of patients with each same mutation previously described. Patient 4’s mutation although published did not have phenotype information for comparison. Variants in Patient 5 and Patient 6 have been submitted to ClinVar (22) without any detailed phenotype descriptions. It is important to note that individual differences in clinical manifestations can occur even with the same genetic variation.

LoF variants include an early stop-gain, indel frameshift or splice-site disruption resulting in truncated protein and reduced or abolished NaV1.6 function.(23) Missense changes causing GoF is the most common pathogenic mechanism for neuronal hyperexcitability and seizures. LoF is associated with cognitive impairment, movement disorders, and autism with or without seizures.(24) The clinical manifestations of *SCN8A* encephalopathy are likely reliant on the degree of GoF or LoF.(25, 26) GoF phenotypes include mild to severe epileptic encephalopathy. There are a few reported cases of benign infantile seizures with mild gain of function too.(27) We identified two GoF and a LoF variant with experimental evidence and three variations with unknown functional consequences. The electrophysiological analyses performed on Patient 4, LoF *SCN8A* variant (p.Cys324Tyr), offer valuable insights into the pathogenesis of *SCN8A*-related disorders. By characterizing the functional consequences of this variant, we provide evidence supporting its role in altering neuronal excitability and ion channel function. This information could potentially inform the development of targeted therapeutic strategies aimed at modulating ion channel activity to alleviate symptoms and improve patient outcomes.

In terms of the *KCNQ3* variant in Patient 4, this variant was found to be a conserved amino acid and all *in-silico* analyses suggest the variant has deleterious impact; however, the variant is novel and remains a variant of uncertain significance. Functional validation has not been performed. Pathogenic variations in *KCNQ3* have been associated with benign familial neonatal and infantile seizures (OMIM 121201).(28, 29) Individuals are typically normal and grow out of their seizures, usually without any neurological sequalae in adulthood. More recently *KCNQ3* mutations are identified in patients with neurodevelopmental disorders and abnormal EEG.(30) Furthermore, alterations in this gene have been reported to act as risk factors for complex diseases including other epilepsy types and autism spectrum disorder. Sands *et al.* delineated an electroclinical phenotype in 11 patients with 4 different heterozygous *KCNQ3* GoF variants. Most of them did not have clinical seizures.(30) Patient 4 had EEG abnormalities with only possible clinical seizures which could plausibly be due to complex underlying molecular mechanisms involving *KCNQ3* and *SCN8A*.

Many early onset neurological diseases are now known to have a molecular basis. A genetic diagnosis can have strong implications for prognosis and treatment of epilepsy.(31) Assessments of how often a genetic diagnosis has clinically actionable implications vary from 20–60%.(32, 33) These comparisons highlight the variability in clinical presentations, epilepsy diagnoses, and genetic diagnoses among the patients with *SCN8A* pathogenic variations.

Intellectual disability, epilepsy, behavioral abnormalities, and movement disorders belong to a complex set of conditions with both monogenic and multifactorial etiologies. Clinical overlap between heterogeneous phenotypes, pleiotropy, variable penetrance, and expressivity makes genetic testing a huge challenge in these families. We describe a cohort of *SCN8A*-related disorders in this research work. The results of this study contribute to expanding the clinical and genotypic spectrum of *SCN8A*-related disorders. By identifying three novel variants in *SCN8A*, we have enhanced our understanding of the genetic landscape associated with these disorders. The observed variability in clinical presentation further emphasizes the complex nature of *SCN8A*-related disorders and highlights the need for personalized approaches to diagnosis, treatment, and genetic counseling. The functional data for p.Cys324Tyr confirms causation in *SCN8A*-related disorders.

## Conclusions

In conclusion, our study adds to the clinical and genotypic spectrum of *SCN8A*-related disorders by identifying novel variants and characterizing the functional consequence of p.Cys324Tyr. These findings underscore the importance of genetic testing in the diagnosis and management of individuals with *SCN8A*-related disorders. The mechanistic insights gained from this study may guide the development of targeted therapeutic interventions to improve patient care and outcomes in this heterogeneous group of disorders. Further research is needed to elucidate the precise mechanisms underlying *SCN8A*-related disorders and to identify potential therapeutic targets for intervention.

## Data Availability

Clinical case summaries are available in the Additional file 1. The datasets generated and analyzed during the current study are available from the corresponding author on reasonable request.

## Abbreviations

OMIM: Online Mendelian Inheritance in Man
LoF: Loss-of-Function
GoF: Gain-of-Function
VUS: Variant of Uncertain Significance
EEG: Electroencephalogram
HEK-293: Human Embryonic Kidney cells
SUDEP: Sudden Unexpected Death in Epilepsy
LGS: Lennox-Gastaut Syndrome
WT: Wild-type
